# The impact of patients as trainers on registered nurses’ patient engagement in primary care clinics: a qualitative study

**DOI:** 10.1186/s12875-023-02210-6

**Published:** 2023-12-13

**Authors:** A. Morin, Y. Couturier, M-D. Poirier, V. T. Vaillancourt, S. Massé, A. D. Tardif, M-E. Poitras

**Affiliations:** 1 Department of Family Medecine and Emergency Medecine, Université de Sherbrooke, Saguenay, Canada; 2 Centre intégré universitaire de santé et de services sociaux du Saguenay–Lac-St-Jean, Saguenay, Canada; 3 CRMUS Research Chair On Optimal Professional Practices in Primary Care, Saguenay, Canada; 4 Department of Social Work, Université de Sherbrooke, Sherbrooke, Canada; 5 School of Nursing, Université du Québec À Chicoutimi, Chicoutimi, Canada

**Keywords:** Patient-centered-care, Nursing, Patient engagement, Practice development, Continuous professional development, Train-the-trainer, Primary care clinic

## Abstract

**Background:**

In Canada, primary care is usually the front door to health care for people with health issues. Among these primary care services are primary care clinics (PCC), where the competencies of registered nurses (RNs) are needed. However, nursing practice in PCCs is variable and sometimes suboptimal from one PCC to another. In 2019, the Quebec Ministry of Health and Social Services deployed a practical guide for RNs practicing in PCCs. This guide was intended to support best professional and interprofessional practices and enhance the quality of services offered according to a physical-social vision of care, interprofessional collaboration and partnership with the patient. The *Formation de formateurs en première ligne* (F2PL) project team developed a train-the-trainer educational intervention to support RNs in assimilating the content of this guide. This educational intervention is uncommon because it includes patients as trainers (PTs). PTs developed and provided andragogic content about patient’s experience to enhance patient engagement.

**Objective:**

To describe the impacts of the educational intervention provided by the PTs in nurses’ patient engagement practices in PCCs.

**Methods:**

A descriptive qualitative approach was used to describe in-depth changes in RNs’ practices. Individual interviews were conducted with 10 RNs and 3 PTs to explore the changes in RNs’ practice and the barriers and facilitators to adopting this new practice. An inductive and deductive thematic analysis was carried out according to a conceptual model of patient engagement (the Montreal Model), and emerging themes were condensed into propositions. To ensure credibility, a peer review was conducted with the F2PL team, which includes a patient co-leader.

**Results:**

The educational intervention provided by PTs has impacted RNs’ practice in 3 ways: awareness or reminding of general principles, updating commitment to already known principles and enhancing the development of new professional skills.

**Conclusions:**

PTs could effectively support the RNs’ motivation to use patient engagement practices in primary care.

**Supplementary Information:**

The online version contains supplementary material available at 10.1186/s12875-023-02210-6.

## Background

In Canada, primary care is the front door to health care for people with health issues [1]. Faced with a growing rate of chronic diseases and an aging population, registered nurses (RNs) working in primary care practices (PCC) support patients in managing their chronic conditions every day [2–6]. Established in 2001 in the province of Quebec, primary care clinics comprise family physicians who collaborate with RNs and other professionals (e.g. social workers, pharmacists, psychologists) to provide care and access to care [5, 7]. However, even if those PCCs have existed for over 20 years, a lack of sufficient guidelines regarding the role of RNs has been observed [5, 8]. This insufficient guidance leads to suboptimal practices [8–10]. The literature indicates that some RNs have developed their practice alone, leading to nursing roles and services varying from one PCC to another [8–10]. When coupled with a response to patients' needs that may be inadequate, the variability of nursing practices in Quebec's PCCs can thus be detrimental to the comprehensive response required for adequate chronic disease management [8, 10]. Those issues, related to a lack of guidance, have been observed across provinces of Canada [5, 11, 12].

In response to this clinical issue, in 2019, the Quebec Ministry of Health and Social Services developed a practice guide to standardize the practice of RNs in PCCs [13]. This guide is composed of three sections: 1) information regarding the operation of PCCs; 2) the expected role for RNs in PCCs, and 3) interdisciplinary collaboration in PCCs. Intending to support professional practices and interprofessional collaboration, the ultimate purpose of this guide was to enhance services offered while integrating a vision of patient engagement [13]. Patient engagement is defined as the participation and involvement of the patient in his or her care according to what he or she wants and is able to do, in partnership with his or her healthcare provider and integrating personalization, access, engagement and therapeutic alliance [14]. Poitras & al., [15] developed a train-the-trainer intervention (*Formation de formateurs en première ligne*, hereafter F2PL) that included clinical trainers and patients as trainers (PTs) to support the implementation of the RNs’ guide and patient engagement approach. This educational intervention integrates PTs’ perspectives in the professional practice development of RNs in PCC by supporting them in integrating the founding principles outlined in the guide. F2PL is a patient oriented research initiative co-lead by a team of researchers (MEP, YC), clinician/decision-maker (SM) and patient partner (MDP). The team worked according to the Canadian Institutes of Health Research recommendations for patient-oriented research [16]. Patient partner involvement in the study is reported in research protocol publication [15] and according to Guidance for Reporting Involvement of Patients and the Public [17].

Little is known about PTs in the context of educational intervention, and there is a need for more research on the impact of PTs in continuing education for heathcare professionals. It is already known that patients have been integrated into different contexts of training to educate, such as mental health, human immunodeficiency virus, chronic obstructive pulmonary disease, geriatrics and spinal cord injury [18–23]. For most continuing educational interventions, PTs’ involvementt is limited to testimony about their lives, their experiences with illness and health services [18, 20–23]. However, Fraser et al. [19] go beyond the testimonies of patients in a mental health educational intervention to increase patient engagement in care and service planning by including them as complete trainers within a team of clinical trainers. Despite the variable description of the PTs’ role between authors [18–23], positive impacts have been reported. Including PTs in educational interventions intended to induce a more remarkable behavior change among clinicians [18–22], modulate practice in partnership with the and patient, improve clinicians’ understanding of patients' needs and how to support them in their care [18, 20–22].

**Fig. 1:**
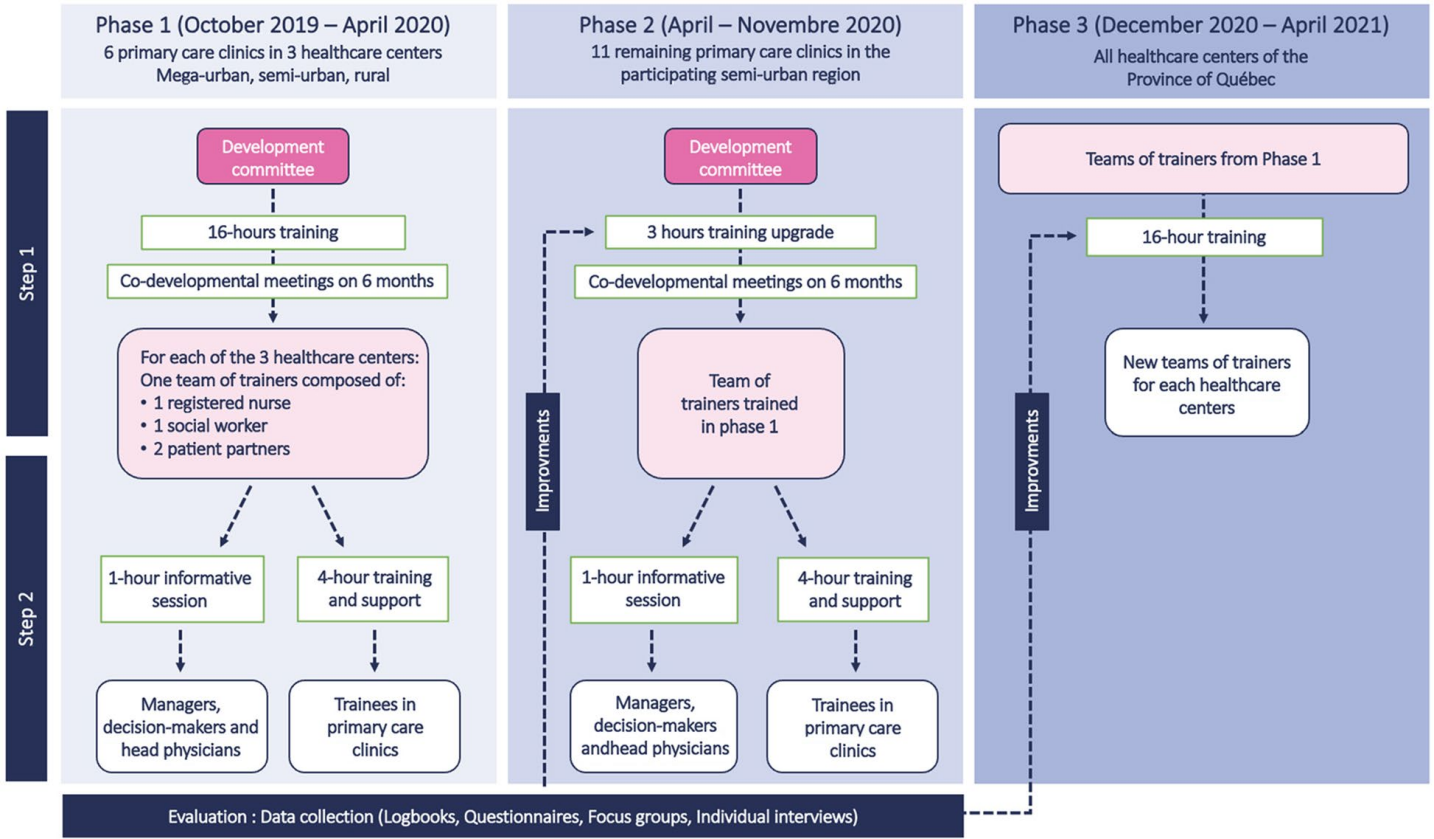
F2PL’s phases. Illustration from F2PL’s phases by Poitras et al. [15], Co-design, implementation, and evaluation of an expanded train-the-trainer strategy to support the sustainability of evidence-based practice guides for registered nurses and social workers in primary care clinics: A developmental evaluation protocol. *BMC Primary Care*, *23*(1), 84.CC-BY

F2PL aims, among other things, to train RNs to enhance their professional and interprofessional practice by engaging patients [15]. The research team trained PTs and clinician trainers to train RNs in PCCs [14] thereafter. The educational intervention explored different themes, including patient experience and partnership, primary care, scopes of practice, and interprofessional collaboration (Table 1). PTs offered content to RNs about health literacy, patient engagement approach, care and chronic disease complexity. F2PL has three phases which are presented in Fig. 1. The present study was part of phase 2 and focused on RNs’ patient engagement approach after the educational intervention. Data were collected along the main F2PL study to reach the following objectives. The complete intervention was described elsewhere [15]. In the present paper, we are taking a step further and relating the data to a patient engagement framework [24, 25]. No study has previously analyzed data using a specific patient engagement framework [18–23]. Using a patient engagement framework provides a better understanding of the PTs’ role that goes beyond life stories and involves the patient as a full trainer. This is how we aim to describe the impacts of the educational intervention provided by the PTs on nurses’ patient engagement practices in PCCs.

**Table 1:** Enhanced Train-the-Trainer intervention content

Module	Overview	Teaching tools used
**Introduction Module**	Host and introduction of participants and trainers. Presentation of the context that led to the deployment of the clinical practice guidelines in Quebec. Objectives of the guides and contextualization of the training of trainers in a perspective of interprofessional collaboration	• Reflexive exercise • Reading • Reading of clinical practice guidelines • Interactive survey • Association game • Clinical cases • Steered discussion • Myth Buster game • Videos • Role-playing • Toolbox • Quiz • Testimonials • Hands-on session • Group discussions
**Module 1**: Andragogy and clinical coaching in the Context of PCCs	Development of skills and confidence to train and coach clinicians. Presentation of different pedagogical strategies	
**Module 2.1**: Primary care and Role of PCCs in care service trajectories	Acquisition of knowledge about front-line services and PCCs to better support clinicians in the change in practice proposed by the deployment of clinical practice guidelines	
**Module 2.2**: Scope of Practice of the registered nurse and social worker in primary care clinics	Improved knowledge of the practice field of clinical nurses and PCC social workers to better support clinicians in developing expected professional practices	
**Module 2.3**: Interprofessional Collaboration in primary care clinics	Acquisition of strategies to accompany health professionals in the development of collaborative practices. Explanation of benefits and added value	
**Module 2.4**: The patient experience	Presentation of the approach in partnership with patients and relatives and valorizing patients' experiential knowledge	
**Module 3**: Hands-on application	Presentation of the multi-level approach, easy manipulation of the proposed teaching material, and practical application of teaching strategies	
**Conclusion Module**	A reminder of the essential elements to remember from the training, round table discussion to gather impressions and comments on the training	
**Co-development meetings and coaching**	Individual or team meetings aiming to consolidate learnings or to address emerging themes of need that have yet to be explored in training

The specific objectives are:

1. To describe PTs’ impact on the assimilation of the patient engagement approach by RNs in PCC after receiving the F2PL educational intervention
2. To identify facilitators and barriers to integrate patient engagement in RNs’ practice
3. To describe PT’s role in an educational intervention
4. To identify characteristics that defined PT as an effective trainer according to RNs

## Methods and design

### Design

As Thorne et al. [26] recommended, a descriptive and interpretative qualitative design must be used to understand the clinical phenomena better and inform about clinical practice reasoning. We choose this design to describe and understand PTs’ role and impact on undocumented phenomena [27, 28]. Some articles about PTs previously described were qualitative studies, and authors reported a better understanding of participants’ experiences [18–20].

### Participants and recruitment

In a semi-urban region of Quebec, Canada, three PTs delivered the F2PL educational intervention to 46 RNs from 10 PCCs. Clinical trainers involved in the educational intervention supported the research team in the recruitment by emailing to RNs. This guided the recruitment of a convenience sample but we paid a particular attention to have variability of perspectives through RNs’ characteristics [29]. Inclusion criteria for RNs were: 1) being an RN in a PCC and; 2) having received the F2PL educational intervention. RNs were recruited until saturation of categories was reached [30].

### Data collection

AM, MEP and YC construct RNs’ interview guide using the Montreal Model adapted from Couturier et al. [25] (Table 2), which is, a patient engagement model that has established a continuum of engagement (information, consultation, implication, partnership co-construction) and is easily transferable to the primary care setting. MEP is an RN and Ph.D. primary care researcher and professor,YC is a researcher and professor in a school of social work and, MDP is a patient partner and co-lead of F2PL project. In the PTs’ interview guide, we included relevant themes emerging from studies about PTs to understand better the phenomenon (e.g. overall experience, PTs’ role, mood, perception of intervention impact, acceptability). Both interview guides were validated independently by two team members (YC and MEP), two patient representatives, two RNs working in primary care and were iteratively modified to ensure the richness of data. Interviews guides are available as supplementary files. We conducted face-to-face or virtual (using Microsoft Teams software (Microsoft Corporation, Inc, Redmond- Washington)) semi-structured interviews with RNs and PTs. Interviews lasted between 30 and 45 min for the PTs and between 45 and 60 min for the RNs. They were conducted in December 2022 by AM, a RN and master’s degree student. Interviews were recorded and transcribed. The interviewer (AM) did not know the RNs’ at the time of the interviews, and she introduced herself as a student at the beginning of the interview.The interviewer (AM) took field notes during interviews to ensure credibility and confirmability [31, 32]. Study participants completed an electronic sociodemographic questionnaire at the interview’s beginning or end. Results from this study are reported according to the Consolidated Criteria for Reporting Qualitative research checklist [33].

**Table 2:** Montreal Model adapted from Couturier et al., [25]

Participation level	Information	Consultation	Implication	Partnership and co-construction
**Patient involved in research**	The documentation given to patients about the research	Patients are consulted on research-specific themes	The implication of patient in research	Involvement of patients from the governance to the dissemination of results in research, including research question
**Patient involved in Health policies**	Information Centre for patients and for the media	Focus group on collecting public opinion	Recommendations made by the patient about healthcare priorities	Co-construction of health policies favourable to health with patients/citizens
**Patient involved in Education**	Use of information obtained by patients in education	Individual patient involvement in courses (testify)	Involvement of trained patients with specific tasks	Co-construction of programs and co-teaching with experiential knowledge sharing
**Patient involved in Organization and quality of services**	The documentation is given to patients on their illness	Focus groups on specific themes	Creation of committees with patients	Co-construction of services, program, quality improvement project
**Patient involved in professional educational intervention**	Unmobilized patient	Patients consulted on their expectations	Patients testify about their experiential knowledge	Co-construction of the educational intervention (objective, andragogical strategies and content) and testify about their experiences and contribute to the educational program as a content expert
**Patient involved in support for practice change**	Unmobilized patient	Patients consulted on their expectations	Patients testify about their experiential knowledge and give an appreciation of change	Co-construction of the support change strategy and testify about their experiences and contribute to the support change strategy as a content expert
**Patient involved in direct care**	Patients receive information (diagnosis, treatment)	Patients are consulted on their perceptions	The shared decision about therapeutic preferences	Patients make decisions based on their life objectives and an interdisciplinary intervention plan

Illustration adapted from Couturier et al. [25] *Par-delà le témoignage; les patients partenaires comme acteurs de la formation professionnelle continue en soins primaire*. In: *L’expérience dans l’innovation en santé: modes éphémères ou nouveau paradigme ?-Regards croisés dans l’écosystème de la santé*. IST

### Data analysis

We coded and analyzed data following Braun & Clarke's [34, 35] thematic analysis approach by following these steps Familiarizing Yourself With the Data, Generating Initial Codes, Searching for Themes, Reviewing Potential Themes, Defining and Naming Themes. Familiarizing Yourself With the Data: AM listened each of the interviews recorded to immerse the data and a detailed summary was written up. Generating Initial Codes: A consultant transcribed interviews, and we imported transcriptions in MAXQDA [36]. The transcripts were coded using the coding grid developed by the research team (MEP, AM, YC, MDP) according to literature and Montreal’s Model to structure a thematic analysis regarding RNs’ patient engagement approach. Preliminary coding was done after each interview to obtain theoretical saturation of categories and reached after interviewing 10 RNs [30]. Two coders (AM and MEP) validated initial codes and refined codes. Searching for Themes: a code trees was created, including all inductive and deductive codes and verbatims [37]. Codes were grouped into initial themes by AM. Reviewing Potential Themes: AM presented the code trees including initial themes to the research team. Each theme and associated verbatims were reviewed in a team meeting and some modifications were made. We condensed emergent themes to formulate proposals that answered the research question and we produced the manusccript [38]. Defining and Naming Themes:We discussed all results and defined themes with all the research team members, including a PT (MDP), to ensure the credibility of the analysis [32]. The sociodemographic characteristics of participants were reported with means using Excel. Descriptive statistics were performed using sociodemographic data to describe the samples’ characteristics and facilitate transferability [32].

### Ethics approval and consent to participate

This project has been approved by the Ethics Committee of Centre intégré universitaire de santé et de services sociaux (CIUSSS) of Saguenay-Lac-St-Jean (SLSJ). It granted ethics approval on July 24th, 2019, under the reference 2019–037. In December 2022, an ethical amendment has been approved by the Ethics Committee at the CIUSSS of SLSJ to do this study. All participants signed an electronic informed consent form to participate in the study before the interviews. All research data have been anonymized, and stored on a secured server; files will be stored in a locked filing cabinet. All methods followed the relevant guidelines and regulations (Declaration of Helsinki).

## Results

Semi-structured interviews were conducted with ten RNs (*n* = 10) employed in 9 different PCCs from a semi-urban region. All RNs were female, French-speaking and Canadian. They had between 1 month and 14 years of work experience in PCCs. All three PTs involved in the educational intervention agreed to participate in the study and were aged between 30 and 59. RNs’ and PTs’ sociodemographic details are presented respectively in Tables 3 and 4.

**Table 3:** Characteristics of registered nurses

Characteristics	Registered nurses N (%)
**Gender**	
Female	10 (100)
Male	0 (0)
**Age**	
30–39	4 (40)
40–49	6 (60)
**Language**	
French	10 (100)
**Country born**	
Canada	10 (100)
**Education**	
Bachelor	8 (80)
Master	2 (20)
**Years in PCC**	
< 1	2 (20)
1–5	5 (50)
6–10	1 (10)
11–15	2 (20)

**Table 4:** Characteristics of patient trainer

Characteristics	Patient trainers (*N* = 3) N(%) >
**Gender**	
Female	2 (66.66)
Male	1 (33.33)
**Age**	
30–39	1 (33.33)
40–49	1 (33.33)
50–59	1 (33.33)
**Language**	
French	3 (100)
**Country born**	
Canada	3 (100)
**Education**	
Diploma of professional study	1 (33.33)
Master	2 (66.66)

Codes from the qualitative analysis have been grouped into themes to answer the different research objectives [35]. In the next section, we will discuss the following themes: Impacts of educational intervention by PTs on RNs’ patient engagement practice, PTs’ role in the educational intervention and characteristics that define PT as an effective trainer according to RNs.

### Theme 1: Impacts of the patient as trainers' educational intervention on registered nurses’ patient engagement practice

The impact of the educational intervention by PTs on the RNs’ practice in PCCs was separated into three effects: 1) awareness or reminder of known principles, 2) updating the commitment to the principles already known, and 3) development of new professional skills**.** The objective “To identify facilitators and barriers to integrate patient engagement in RNs’ practice” was included in this section as it affects the integration of patient engagement into nursing practice. We define the awareness of RNs by their ability to recall previous knowledge on the principles of patient engagement. Updating their commitment to the principles previously acquired signifies that the RNs review acquired content and, after that, update or modulate their knowledge and commitment. The development of new professional skills describes concrete, observable changes in the clinical practice of clinicians. These follow a continuum leading to professional development.

### Awareness or reminder of known principles

Many trained RNs reported that the educational intervention had raised their awareness to use better centered-care practices with patients. They especially mentioned that RNs suggested being more empathic towards patients with health issues. Many RNs said being more sensitive to the complexity of patient appointments, for instance, how long it takes to get ready for an appointment when living with health issues after being trained by the PTs.

*“I found it really relevant to our practice because we forget to put ourselves in the patient’s shoes, I had never realized that it could take that long for someone to get ready in the morning to leave”* (RN9)

Trained RNs also reported being sensitized by PTs about judgment. For instance, when patients do not attend their appointments, RNs said they are now more aware of their behavior, especially when the turnover puts pressure on them, which can promote judgments. Training by PTs has led to the consciousness that patients are humans as well, i.e. they have a life of their own and a living context of their own, so their actions should be contextualized.

*“ […] it is easy to judge the person who doesn’t show up, to get out of the context of our work schedule, of appointments and of being in a hurry, and that we are wasting time, but to try to, this is part of getting out of the context, that sometimes we drive a little too hard, we work with humans, not with machines.”* (RN7)

### Updating the commitment to the existing principles

The educational intervention led by PTs enabled them to update RNs’ commitment to existing principles by motivating them to improve their practice. Trained RNs have been made aware of the need for better practices regarding patients living with health issues and are now more inspired to modify their practice accordingly. Some RNs said they are ready to move from theory to action, particularly in grouping patients’ appointments.

*“ I would even tell you for the whole team that we have learned to try to put our appointments at the same time, for example, with the doctor, with the social worker, to group a little bit.”* (RN1)

They also want to improve partnering and consulting with patients by questioning them about their objectives regarding health issues. They were ready to update and enhance sub-optimal practices. They mentioned that they want to work on some aspects while needing improvement.

*“ […] I try to correct myself like things like that, I am not yet top, I have just started, sometimes I forget, then I fall back in my old shoes, but I try to do more and more, and it is more automatic.” (*RN9)

One RN said that she reviewed known principles, realizing that she needed to update her practice by asking the patient more questions about their perception of health issues.

*“I became aware again of what I was already doing, that I had probably relaxed because we also did many telephone follow-ups. In telephone follow-ups, it is different the verbal but also physical of the patient compared to what we see, so that probably, I think that I had relaxed a little bit so that, which brought me back to re-question them more and then to see more what their perception is”* (RN8)

Involving PTs in educational intervention brings a readiness to query the spheres surrounding the patient. For example, RNs mentioned they want to question patients about their environment to understand better the barriers they face and the impact of their illness on their daily life. They want to expand their span of questioning to improve their nursing assessment. They want to ask more questions about psychological elements and go beyond the physical aspect.

*“ […] they also challenged us, to question beyond the visual, and sometimes at the psychological level, how do they feel, and you know, sometimes, we feel it, we see it, but sometimes it is perhaps a question we could ask, is everything okay today? It is pretty broad; we can go particularly to the level of morale, maybe go deeper into how they might be feeling.”* (RN3)

Finally, reviewing some general principles allowed RNs to ensure that their approach to the patient is consistent with what is being promoted by the practice guide. The update provided by the educational intervention also encouraged RNs to continue good practices regarding patient engagement, especially when patients are distressed.

*“There was a lady who had difficulty breathing, she was not well in the waiting room, I took her to a room, I don’t do that anymore, but it was something I was already doing, but I think it’s like a plus, to say that I’m doing my job well”* (RN10)

### Professional development of new skills

A reminder and update of the principles of patient engagement naturally led to developing new skills. RNs reported substantial practice changes related to patient engagement after the educational intervention. Many RNs mentioned that they ask more questions about patients’ needs, expectations and experiences. One RN mentioned now asking patients more about their illness perception and experience of care:

*“I take more time to validate with them how they are doing and then what I can do for them today, I tell them that we are seeing each other today for their diabetes or asthma or whatever, how are you doing, is there anything else I can do for you, I validate with them if they have another need”* (RN 10)

Other participants mentioned they had improved their approach to shared decision-making with patients having complex needs. Some RNs reported that, since being trained by F2PL, they have been trying to further the principles of shared decision-making during meetings with patients with health issues. For example, an RN asked a patient about his solutions to make diabetes less unstable, and she rarely did that before. She was then more skilled in building a care plan, including the patient’s solutions and presented different options to support self-management. In the end, she enabled the patient to make a decision:

“*[…] diabetes is unstable […] instead of just saying – We will do this, we will do that, I give them really, I try to present them the options, before, I was like not really doing that, I was more evaluating them and then telling them what they could change, so instead, I ask them first if them they see anything that could make things less unstable at the moment, then if they don’t really see it, well then I explain to them what the options are, so I explain to them accordingly let’s say diet, physical activity, anxiety, medication, so I explain to them what the options are, and then I give them the decision*” (RN9)

Another RN reported better skills in the patients’ involvement in their care and treatment decisions. She puts the patient as a stakeholder in the decision-making process, giving more weight to the patient. As a result, RNs better understand how to engage patients in shared decision-making:

*“ I ask my patient at the end, too, and I ask him at the end of our plan is correct, if my plan is this, this, this, do you agree with the plan, and then about a previous follow-up, I’ll do that more too, I’ll validate it too – When do you want to meet? I’ll make a plan, I’ll make him a stakeholder in this finally, of this meeting, that it’s not just that he comes to see me once a year, him if he needs to come and see me three times a year, well it can be three times a year*.*”* (RN10)

Changes in RNs’ self-reported professional skills mainly focus on the consultation and implication aspects of the Montreal Model’s patient engagement continuum. RNs collect more of the patient’s perspective by questioning them about their experiential knowledge, environment and psychological aspects related to the consultation in direct care. They also better engage patients in shared decision-making by giving a voice to patients.

However, RNs stated that different factors positively or negatively influence optimal patient engagement. Barriers and facilitators reported by RNs to achieving an optimal patient engagement approach are presented in Table 5. Facilitators for which respondents placed importance were patients and family skills that help to build a positive climate relationship.

*“A patient who is like open, when the patients arrive with their spouse also, we see that their loved ones are involved and that they want to help too, I think that is motivating for the sick patient and us as well, we say to ourselves “ My God, he is going to be well surrounded.”* (RN1)

**Table 5:** Barriers and facilitators to achieving an optimal patient engagement approach

Barriers	
Technological • Phone follow-ups	*“I had probably relaxed because we did many telephone follow-ups too; in telephone follow-up, it's different, the verbal but also the physical of the patient and what we see”* (RN8)
Organizational • Turnover context imposed by the health care system	*" …it's easy to judge the person who doesn't show up, to get out of the context of our work schedule, of appointments and of being in a hurry, and that we are wasting time, but to try to, this is part of getting out of the context, that sometimes we drive a little too hard, we work with humans, not with machines.”* (RN7)
Experiential • Past negative patient experiences can negatively influence their relationship	*"Well, that’s it, like I said, maybe past experiences that have been like more negative with other professionals (…) patients who have not felt listened to, patients who have not felt well taken care of, or patients who feel like they are repeating their story 56 million times, and then they feel like—Look, I just told it, it's been 2 min, to the other person, you're not talking to each other. I think that makes the patient feel good or not."* (RN1)
Facilitators	
Patient and family skills • Patients motivated to manage their health issues • Involvement of families in care	*" What is facilitating is that a patient who shows interest, that’s sure that it’s always fun, we see that he is motivated, there are patients who arrive and who are previously aware of their health problem, who have gone to read about it, who are, that is certainly facilitating, and it is also motivating for us too.”* (RN1) *"A patient who is like open, when the patients arrive with their spouse also, we see that their loved ones are involved and that they want to help too, I think that is motivating for the sick patient and us as well, we say to ourselves “ My God, he is going to be well surrounded.”* (RN1)
Organizational • PCC context	*“I think that we have the time here, in addition, in the PCC, to be able to allow ourselves this, all the beautiful approach that we can have and then we have time, and versus me, the urgency that I have the time to do nothing of this”* (RN1)

On the other hand, an example of barriers to patient engagement practices reported was telephone follow-ups, as they bring a physical barrier.

*“I was still going to talk about it with the doctor, and I offered him to come to the office here so that he could meet me because I felt that over the phone, sometimes, there is always a bit of a barrier”* (RN1)

### Theme 2: Patient trainers’ role in the educational intervention

Data collected from PTs explained their role throughout the educational intervention. Since the role of the PTs is innovative, the Montreal Model adapted by Couturier et al. [25] allowed us to categorize PTs’ involvement along the continuum of patient engagement in the professional educational intervention (Table 6). At the beginning of the educational program, a PT noted difficulty fully embodying its role because some RNs were shy and weren't sure why patients came to educate them. On the other hand, as time progressed, the PT was able to take on a more active role. Here is how the work of the PTs has led to the above impacts:

**Table 6:** Patient trainers*’* role in educational intervention according to Couturier et al., [25]

Level of patient engagement in professional educational intervention according to the Montreal Model	Patient trainers’ role
Implication	• Sharing experiential knowledge • Educating RNs about the complexity of patient care
Partnership	• Contributing as an expert on patient’s experiential knowledge • Transmitting educational intervention and andragogical content to learners

PTs reported sharing experiential knowledge by testifying about healthcare experiences and living with health issues. They also trained RNs about the complexity of care related to the restrictive elements that influence patients’ well-being:

*"Let us go into the restrictive elements, you know, the elements to be taken into consideration for the patient, just for travel, for example, how do we feel during a meeting, we can have stress, we do not understand everything"* (PT1)

More originally, PTs contributed as content experts with their experiential knowledge of living with health issues. They affirmed having their knowledge just like RNs have theirs. They reported feeling like collaborators bringing new knowledge beyond simple testimonials and providing andragogical content about the patient experience.

*" […]we present a PowerPoint with scenarios, and then we try to put a good atmosphere for an exchange again and to explain our patient background a little bit to open the discussion about a patient is not just a patient; it is someone exhausted from their day, it is someone with problems sometimes”* (PT3)

They mentioned using methods to support the change of practice adapted to adults and valued RNs in their role with the patient.

*" With adults, we raise awareness, we will never say to someone " Well, that is not the right answer you gave me. "We will try to get the person's head around it, but we will not try to tell them " Well, you are in the field completely " because we know we'll put them on edge, and then it won't work."* (PT2)

Finally, one PT added having the responsibility to support RNs' change in practice by sharing more than experiential knowledge. For PTs, it was not only the transmission of a simple testimony but a collective role in supporting patients receiving care in primary care. PTs also said they have a role in representing all patients living with health issues.

*"I felt that I had a responsibility towards all the people who gave us their experiential knowledge as well because it's not Patient Trainer who tells his life story, I help train people on a change of practice that is based on the lives of many people, it's a responsibility that we also have towards these patients.”* (PT 2)

According to the Montreal Model adapted by Couturier et al. [25], those roles are related to the level of implication and partnership. This implies that the PT’s role is about testifying experiential knowledge, presenting andragogical content and methodologies, participating in clinical coaching and being an expert in experiential knowledge.

### Theme 3: characteristics defining PT as an effective trainer according to RNs

RNs reported the following elements as critical to define an effective PT in an educational context. RNs have highlighted the elements that must be present to ensure the effectiveness of PTs’ role. Table 7 presents the necessary characteristics the PT must have or acquire.

**Table 7:** Characteristics the patient trainer must have or acquire to be an effective trainer

	**Characteristics of an effective patient trainers**	***Verbatims***
1	Ability to tell experiential knowledge and make it clear	*"(…) the patient trainers ****talked to us about their experience, their good experiences**** and ****their not so good experiences****, (…) it's always ****appreciated to see a little the vision of the patient**** versus u****s as professionals****.”* (RN1)
2	Capacity to interact with registered nurse	*"I found it very interesting to ****talk with the client but, you know, not in a care contex****t but in a context just like that of research and then to know how the client can feel”* (RN4)
3	Ability to convey andragogical content and methodologies effectively and accurately	*"(…) yesterday I had another patient partner in another context, it had the same effect****, it's so tangible****, so I think that the patient partner, we have to go towards that, and it has been a few experiences of patient partners, I had another one in a context as well, and me it remains, ****the stories of the people, we work with the people, so I think that as a health care staff, we have this sensitivity that a true story in an educational intervention context will mark us**** more than an educational intervention practically online, that we will do by ourselves, we will listen, but put a testimony in there and then it seems that, but in any case, for me, an online educational intervention versus a testimony of a person who lived it, there I understand them, it just scanned, so for me****, I like the patient partners****. They are in the ****best position to share their experiences so that we can improve**** on what they have experienced”* (RN7)
4	Being actively engaged in the role of PT in educational intervention	*"What impressed me was ****especially their involvement,*** I think. *They are ****motivated****, they want to, but I find that the fact that they are so motivated is because they have lived through things that they probably found difficult, surely there were good sides as well despite everything”* (RN8)
5	Create an atmosphere favourable to reflexivity	*"Well, ****it makes them aware of how they can feel****, but on the other hand, I am also a patient at certain times, so it's not unknown how they can feel. I don't have a chronic illness, but I have had problems where I had to consult so that you can understand, but it was. Still, it was touching; it didn't leave you indifferent.”* (RN2)

All RNs appreciated the experiential knowledge transmission and reported that it must be integrated into an educational intervention to understand patient and professional views better. RNs reported many enablers to facilitate content PT’s appropriation, such as having the opportunity to interact in a different context from that in which the nurse provides care to the patient. According to RNs, it helps to integrate the educational intervention because the PTs’ content included true stories, and it marked them. RNs also reported that the PTs must be able to convey andragogical content and methodologies effectively and accurately because the contents offered by PTs enable better understanding. After all, PTs give examples, and it is more interactive. RNs’ said that PTs must be motivated by their trainer role because it demonstrates their determination to improve patient’s heathcare. Finally, RNs mentioned that PTs should create a positive atmosphere during educational sessions and support RNs in introspection because RNs can be patients anytime. These data allow us to identify better what RNs expect when receiving educational intervention from PTs.

## Discussion

Our study described the impacts of the educational intervention provided by the PTs on nurses’ patient engagement practices in PCCs. Specific objectives were to describe PTs’ effect on the assimilation of the patient engagement approach by RNs in PCC, to identify barriers and facilitators to achieving an optimal patient engagement approach, to describe the role of PTs in the educational intervention and to identify characteristics defining an effective PT according to RNs. As far as we know, it is the first study to explore this subject with a cohort of RNs longitudinally in the F2PL project. Overall, we found that 1) Educational intervention involving PTs supports the inclusion of patient's perspectives to allow RNs to focus on the real needs of the patients; 2) PTs effectively support the RN’s motivation to change by adopting better practices through patient engagement; 3) Difficulties encountered about this new patient's role are similar to those for other types of patient involvement and; 4) Montreal Model is a relevant model in the evaluation of the patient engagement continuum in an educational context. These results lead us to the following observations.

We found that educational interventions involving PTs support the inclusion of patients’ perspectives to allow RNs to focus on the real needs of the patients. What we mean by real needs, is what matters the most for the patient according to his/her reality/perception. Our results corroborate existing literature [18, 20–22] findings that integrating patients into the educational intervention for health professionals brings lasting benefits on several levels, particularly in the therapeutic patient-professional relationship. Our study identified several PTs' impacts on RNs’ practice. The RNs attributed those impacts to the roles of the PT in the educational intervention. This result corresponds to the impacts Kroll et al. [21] observed. Indeed, these authors reported that health professionals appreciate the testimonies of PTs and state that the patient’s perception leads to a broader vision of care. This observation was also described by McCreaddie et al. [22], Foster et al. [20], Kroll et al. [21] and Chambers et al. [18], who suggest that the life stories of PTs help clinicians understand and support them in their realities, particularly with symptom management, medication management, and living with the obstacles of the disease. However, health professionals sometimes perceive the PTs’ approach negatively [22]. When a PT trains a cohort of RNs longitudinally (as in the present study), a bond of trust and mutual engagement seem to be created, as well as a recognition of the experiential knowledge and the added role of the PTs’ [15], at this stage, it's difficult to say whether educational intervention added value beyond testimonials. However, our results show that RNs appear more likely to take action when being trained by PTs compared to educational intervention, where patients have a limited role. It is possible to hypothesize that the PT's role, going beyond testimonials, would benefit changes in professional practice. In contrast, the role of the usual PTs seems more focused on raising awareness. Further studies will be necessary to confirm this hypothesis.

Our results also demonstrated that PTs effectively support the RN’s motivation to change by adopting better practices through patient engagement. We described clinicians as more motivated to improve practices by engaging patients in care when following educational interventions, including PTs. For example, RNs are more likely to be open to patients' needs, ask them questions, understand their situation, and be more aware of their judgment and the importance of avoiding them. A person-centred approach and modulating an RN intervention according to the patient's needs are also part of best practices in primary care and health issues management [39, 40]. The story-telling approach to education and the different types of andragogic activities that supported patient perspectives and realities have effectively improved nursing practice. Foster et al. [20] state that health professionals develop empathy when hearing the story-telling of PTs living with chronic obstructive pulmonary disease. This supports motivation for changes toward patient-centred practices [20]. We also found that the educational intervention offered by PTs helped RNs to update their practice with patients. This is consistent with Chambers et al. [18], that reported clinicians were more confident interacting with PTs with mental health problems, which motivated them to adopt new practices. In addition, the educational intervention allowed the participating RNs to become more aware and change some judgments about patients, especially those who do not show up for their appointments and are not involved in their care. McCreaddie et al. [22] reported that clinicians were more sensitive to hasty judgments about the HIV population and that there is a need to challenge clinicians’ attitudes after being trained by PTs. Our study confirms that the content presented by PTs allowed RNs to put their behaviours into perspective, resulting in a better understanding of the patient as a whole, which may contribute to improving the quality and satisfaction of patient care.

Our study demonstrated that the new patient's role coincides with the difficulties recognized by other types of patient involvement. The potential for enriching the role of PT is challenged by the difficulties recognized for other roles patients may have. We highlighted some reluctance at the beginning of the educational intervention, but with time, the RNs modified their behavior and acknowledged the PTs as professionals of their experiential knowledge. This concern was also raised by Codsi et al. [10], who demonstrated that involving patients in continuous quality improvement committees in primary care may cause discomfort for professionals by confronting their ideas with patient's ones. Codsi’s study also stated that professionals are afraid to show their imperfections, fearing distrust in the professional. They perceive the patient as a vulnerable being they must care for but agree they must work in partnership with the patient, thus decreasing the hierarchical relationship [10]. Although patient involvement is different in Codsi et al.'s study [10] than in ours, some similarities can be drawn, such as the need to please the patient and greater inclusion.

### Montreal Model is a relevant model in evaluating the patient engagement continuum

Furthermore, the Montreal Model allowed us to classify the results of our study on RNs' practice in the continuum of patient engagement in direct patient care [9]. Following the educational intervention, RNs are found at the consultation level, when they mainly ask the patient about their perceptions, and at the collaboration level, when they claim to have improved shared decision-making with the patient. Considering that the majority of the studies reviewed engaged the PTs only in transmitting their experience in the form of a testimonial and did not classify the role in a patient engagement continuum, we believe that the enhanced role of the PT in our study generates a more critical impact in the continuum of patient engagement of the Montreal Model and provides a better understanding of the role. Our study, therefore, shows the relevance of enriching the role of PTs and going beyond the logic of testimony by positioning the PT as an expert and a trainer with healthcare professionals learning in their work environment, which distinguishes the present study from the authors we have just cited [18, 20–22]. As we become more familiar with the role of the PTs, improvements to the Montreal model will be made.

### Implication for clinic and research, strengths and limitations

Our study fills a gap in the literature regarding the impact of PTs on healthcare professionals learning in their work environment. Our study provides scientific data on integrating the patient in a Train-the-trainer intervention in primary care. As PCCs are geographically delocalized entities, it can be challenging to implement training innovations with several RNs simultaneously [41]. According to this challenge, our study showed that PTs could be included in the RNs’ educational intervention even if their PCCs are geographically delocalized. Our study, therefore, adds to the body of knowledge on improving the professional practice of primary care nurses through PTs. It is a unique contribution to patient-centred research and an innovative approach to training RNs in primary care.

From a methodological point of view, the participatory research framework allowed for the inclusion of all stakeholders, including policymakers, researchers, patients, and professionals, to ensure inclusiveness and to meet everyone's needs [42]. The RNs sample was varied to ensure maximum variation in data and an opportunity to ensure transferability to a similar setting [32]. However, the results of this study came from a specific region with its context, which may affect the transferability of the results to another context or environment [32].

Further studies will be needed to identify PTs' impact on patient care quality because we could not collect data from patients due to the impossibility of recruitment because RNs had a heavy workload, and some were moved to other locations during COVID-19 pandemic. In the future, data must be collected from patients to ensure credibility and explore educational interventions' impacts on patients’ experiences and outcomes [32]. Although we did not collect data on patients' reported outcomes and experiences, we used triangulation of data sources, co-coding, and peer discussion, including the patient co-leaders, to ensure the results' credibility [31, 32]. The logbook also made it possible to record all the elements related to the stages of the research project and the lead author's (AM) personal reflections, ensuring the reliability and confirmability of the study [31, 32]. RNs' responses may be influenced by certain desirability, which may undermine credibility, but to limit bias, we have collected data from different sources and methods [32]. We also had several discussions with the research team to ensure the analysis was consistent with the empirical data [23].

## Conclusion

To our knowledge, our study is one of the first to explore PTs’ engagement in educational intervention for RNs in PCCs. Our work sheds light on the potential added value of the PTs in the continuing education of RNs in clinical settings and PCCs. The educational intervention allowed RNs to update their practice with patients and to develop their professional practice of patient engagement. The role of the PTs and the characteristics necessary to incarnate this role also provides insight into their impact on nursing practices. We also noticed that RNs seem more motivated to take action when receiving andragogic educational intervention from the PTs. Further studies will be necessary to investigate how the added value of PTs to continuing education can be sustainable in primary care and nursing practices or to understand the effects on patient outcomes regarding health issues management.

### Supplementary Information

**Additional file 1.**

**Additional file 2.**

## Data Availability

The datasets used and analyzed during the current study are available from the corresponding author upon reasonable request.

## Abbreviations

RN: Registered nurse
PCC: Primary care clinic
PT: Patient as a trainer
TTT: Train‑the‑trainer
