# Supplementary material for: The impact of patients as trainers on registered nurses’ patient engagement in primary care clinics: a qualitative study
Source: BMC Prim Care. 2023 Dec 13;24:265. doi: 10.1186/s12875-023-02210-6 (PMC10717897; doi:10.1186/s12875-023-02210-6)
Supplement: Supplementary file 1 — Additional file 1. [file 12875_2023_2210_MOESM1_ESM.pdf]

Semi-structured interview guide for patient trainers

Thank you for taking the time to speak with me today. First, I'd like to tell you a little about the study. As you may know, F2PL training aims to support the assimilation of the Ministry of Health and Social Services (MSSS) practice guides by supporting the enhancement of professional and interprofessional practices based on a vision of patient engagement. Consequently, the interview aims to describe the training provided by patient trainers and to obtain their perspective on the educational program's effect on clinicians.

We'll ask you questions about the course of the intervention, your role, and your perception of the effects of the intervention. This should take about 45 minutes. I want to record our interview today so I don't miss anything you say. Would that be all right?

Your identity will remain confidential, and your name will never be used. You can end the interview anytime or choose not to answer a specific question. Do you have any questions before we start?

General experience

1. Can you tell us about your experience as a patient trainer for registered nurses?
2. Tell us about your experience as a patient trainer with the research team?

Role of the patient trainer

3. Describe your role with the registered nurses during training?
4. What made it easier for you to act as a patient trainer for the registered nurses?
5. What made your role as a patient more difficult when training registered nurses?

Mood

6. How did the registered nurses you trained welcome the training offered by the patient trainers, specifically during the portion you gave?

Perception of the impact of the training intervention

7. How do you think registered nurses will use the knowledge taught about the patient experience in their practice?
   a. How can you observe this change in their practice?

Acceptability

8. What is your level of appreciation of the Patient Experience training you led?
9. Do you consider that the content taught has helped registered nurses to use patient-centred practices in partnership with the patient? If yes, why? If not, why not?
10. Do you have any recommendations for the success of the patient-trainer role with registered nurses?

Thank you for your participation!
