# Supplementary material for: The impact of patients as trainers on registered nurses’ patient engagement in primary care clinics: a qualitative study
Source: BMC Prim Care. 2023 Dec 13;24:265. doi: 10.1186/s12875-023-02210-6 (PMC10717897; doi:10.1186/s12875-023-02210-6)
Supplement: Supplementary file 2 — Additional file 2. [file 12875_2023_2210_MOESM2_ESM.pdf]

Semi-directed interview guide for registered nurses on the assimilation of the patient engagement approach

Thank you for taking the time to talk to me today. This meeting aims to understand the implementation process of the educational intervention and how professional practices have been deployed since the beginning of the project. We're also interested in understanding what makes adopting professional and interprofessional practices more accessible or difficult.

We'll ask you questions about your professional practice, particularly about direct patient care.
This should take about 60 minutes. I want to record our interview today so I don't miss anything you say. Would that be all right?

I want to tell you that your identity will remain confidential and that your name will never be used, nor will it ever be possible to identify you. You can end the interview anytime or choose not to answer a question. Do you have any questions before we start?

We'll look at an innovative aspect of your educational intervention, integrating patient trainers. We'll look at how the presence of the patient trainer can influence (or not) your practice. There are no right or wrong answers.

Information

8. When you meet a patient with a chronic disease, what do you focus on first?

Consultation

9. How do you validate the patient's understanding of their care and treatment?
a. Give me an example of what you do now.

Implication

10. Tell me about the shared decision-making process with the patient during your consultations?
a. Tell me in detail about the last consultation in which you shared decision-making with the patient.

Partnership

11. How do you support patients in their self-management process?
"A person's ability to manage their symptoms, treatments, physical and psychological consequences and lifestyle changes inherent in living with a chronic condition."

a. Give me an example of how you now support the patient in the self-management process.

12. How does the patient's expertise help you?
a. Give me a concrete example.

13. what makes it challenging to partner effectively with the patient in your practice?

14. In your practice, what facilitates effective partnership with the patient?

15. Tell me about your general experience with patient trainers?

16. Due to the training received, have you adopted any changes in your practice? If so, give me a concrete example.
a. Do you have any other examples of changes?
b. What has changed or not changed your practice about the patient-centred approach?

Thank you for your participation!
